# Reduced hepatic GLUL expression accompanies altered RIP3/MLKL-pathway protein abundance in early cholestatic liver injury following bile duct ligation

**DOI:** 10.3389/fnut.2026.1912372

**Published:** 2026-10-02

**Authors:** Baiyu Yao, Fanhe Xiao, Zhong Tian, Juan Kong

**Affiliations:** 1 Department of General Surgery, Shengjing Hospital of China Medical University, Shenyang, China; 2 Department of Clinical Nutrition, Shengjing Hospital of China Medical University, Shenyang, China

**Keywords:** bile duct ligation, cholestatic liver injury, GLUL, necroptosis-related genes, RIP3/MLKL-related signaling

## Abstract

**Background:**

Obstructive cholestasis is characterized by disturbances in bile acid homeostasis, amino acid metabolism, inflammatory responses, and hepatocellular injury. Although RIP3/MLKL-pathway proteins have been implicated in cholestatic liver damage, their relationship with metabolic alterations remains incompletely understood. This study aimed to identify necroptosis-related genes and associated metabolic-pathway alterations in early bile duct ligation (BDL)-induced cholestatic liver injury through integrated bioinformatics analyses and experimental validation.

**Methods:**

Gene expression datasets GSE29776 and GSE166867 were retrieved from the Gene Expression Omnibus database. Differentially expressed genes (DEGs) and necroptosis-related differentially expressed genes (NRDEGs) were identified. Functional enrichment analyses, protein–protein interaction network construction, least absolute shrinkage and selection operator (LASSO) regression, and random forest analysis were performed to prioritize candidate genes. A murine BDL model was established for validation using biochemical assays, histological analyses, RT-qPCR, and western blotting. Phosphorylated RIP3 and MLKL were evaluated both relative to GAPDH and as phosphorylation-to-total-protein ratios.

**Results:**

Eight NRDEGs were identified, including Tnfaip3, Sqstm1, Chmp2b, Ifngr2, Slc25a4, Ifngr1, Camk2d, and Glul. Functional enrichment analyses revealed significant enrichment in pathways related to metabolic regulation, transmembrane transporter activity, inflammatory signaling, and necroptosis. Machine-learning analyses prioritized Sqstm1, Ifngr1, and Glul as candidates for exploratory characterization. Glul, which encodes glutamine synthetase (GLUL), was markedly downregulated in cholestatic liver tissues. Experimental validation confirmed bile acid accumulation, reduced Glul mRNA expression and GLUL protein abundance, and increased GAPDH-normalized RIP3, p-RIP3, MLKL, and p-MLKL abundance in BDL mice. Both the p-RIP3/RIP3 and p-MLKL/MLKL ratios were significantly increased, supporting enhanced phosphorylation of RIP3 and MLKL relative to the corresponding total-protein levels.

**Conclusion:**

In early BDL-induced cholestatic liver injury, reduced hepatic Glul mRNA expression and GLUL protein abundance occurred concurrently with increased RIP3 and MLKL phosphorylation relative to the corresponding total-protein levels. These findings support enhanced RIP3/MLKL-associated signaling but do not independently establish pathway-dependent necroptotic flux. Further mechanistic validation is required.

## 1 Introduction

Obstructive cholestasis is a common pathological condition encountered in hepatobiliary diseases, including choledocholithiasis, pancreatic head carcinoma, cholangiocarcinoma, and ampullary tumors (1, 2). Persistent biliary obstruction results in bile acid accumulation, hepatocellular injury, inflammatory cell recruitment, cholangiocyte proliferation, progressive fibrosis, and ultimately cirrhosis (3, 4). Despite substantial advances in understanding the pathological consequences of cholestasis, the molecular mechanisms linking metabolic disturbances to hepatic injury remain incompletely understood.

Beyond their classical role in lipid digestion and absorption, bile acids are increasingly recognized as endocrine signaling molecules that regulate glucose, lipid, amino acid, and energy metabolism through receptors such as farnesoid X receptor (FXR) and Takeda G protein-coupled receptor 5 (TGR5) (5, 6). Disruption of enterohepatic circulation during obstructive cholestasis leads to profound alterations in bile acid homeostasis and metabolic reprogramming (7). Accumulating evidence suggests that dysregulated bile acid signaling contributes not only to hepatocellular toxicity but also to oxidative stress, immune activation, and disturbances in nutrient metabolism. Furthermore, because bile acids serve as key mediators of gut–liver communication, disruption of bile acid signaling may contribute to gut–liver axis dysfunction and immune imbalance during cholestatic liver injury (8–10). These findings highlight the importance of metabolic regulation in the pathogenesis of cholestasis.

Metabolic reprogramming is increasingly recognized as an important determinant of hepatic homeostasis and inflammatory responses (11). Among amino acids, glutamine is the most abundant circulating amino acid and plays essential roles in nitrogen metabolism, antioxidant defense, immune regulation, and cellular energy homeostasis (12–14). Evidence indicates that dysregulated glutamine metabolism is associated with inflammatory activation, liver fibrosis, and cancer-related metabolic remodeling (15–17). Glutamine synthetase (GLUL), a key enzyme responsible for glutamine biosynthesis, plays an essential role in maintaining hepatic nitrogen homeostasis and metabolic adaptation. However, the relevance of GLUL expression to glutamine-related metabolic regulation in cholestatic liver injury remains unclear.

Necroptosis is a regulated form of programmed necrotic cell death characterized by plasma membrane rupture, release of intracellular contents, and amplification of inflammatory responses (18). The canonical necroptotic pathway is mediated by receptor-interacting protein kinase 1 (RIP1), receptor-interacting protein kinase 3 (RIP3), and mixed lineage kinase domain-like protein (MLKL) (19–22). Upon activation, RIP1 and RIP3 form the necrosome complex, resulting in RIP3 phosphorylation, subsequent activation and phosphorylation of MLKL, and ultimately plasma membrane disruption. Emerging evidence indicates that necroptosis is associated with both metabolic stress and inflammatory disorders, suggesting possible interactions among these processes during cholestatic liver injury (23, 24).

Immune dysregulation is another hallmark of obstructive cholestasis. Persistent accumulation of bile acids promotes recruitment and activation of immune cells, thereby exacerbating hepatic inflammation and tissue injury (25–28). Previous studies have described macrophage activation and altered inflammatory signaling during cholestatic liver injury (25, 27–30). Although both necroptosis and immune activation have been implicated in cholestatic liver injury, the interactions among metabolic dysfunction, necroptosis, and immune remodeling remain largely unexplored.

In the present study, we integrated transcriptomic datasets from the Gene Expression Omnibus (GEO) database with experimental validation in a murine BDL model to identify necroptosis-related differentially expressed genes (NRDEGs) and associated metabolic alterations. Through bioinformatics analyses, machine-learning approaches, and *in vivo* validation, we sought to characterize concurrent changes in metabolic pathways and RIP3/MLKL-pathway proteins during early cholestatic liver injury. Particular attention was focused on glutamine synthetase (GLUL), a key enzyme in glutamine biosynthesis and nitrogen metabolism. Our findings identify reduced GLUL expression as a candidate molecular feature for further investigation.

## 2 Materials and methods

### 2.1 Data collection and processing

Gene expression profiles were obtained from the Gene Expression Omnibus (GEO) database (https://www.ncbi.nlm.nih.gov/geo/). The GSE29776 dataset included three liver samples collected 24 h after bile duct ligation (BDL) and three sham-operated (SH) controls. The GSE166867 dataset contributed five liver samples collected 24 h after BDL and three SH controls. Thus, 24 h was the common post-BDL interval used for integration across both datasets. Raw expression matrices were integrated and preprocessed using the R packages “limma” and “sva” to remove batch effects, perform probe annotation, normalize expression values, and generate a merged expression matrix for downstream analyses.

### 2.2 Identification of necroptosis-related differentially expressed genes

Differentially expressed genes (DEGs) between BDL and SH groups were identified using the “limma” package. Genes with *Benjamini–Hochberg-adjusted p* < 0.05 and |log2FC| > 0.5 were considered DEGs. Volcano plots and heatmaps were generated using the “ggplot2” and “pheatmap” packages. For heatmap visualization, DEGs meeting *Benjamini–Hochberg-adjusted p* < 0.05 and |log2FC| > 0.5 were ranked by log2FC, and the 20 most upregulated and 20 most downregulated DEGs were selected. Heatmap values represent row-wise standardized expression values (z-scores), whereas the volcano plot displays log2FC and *Benjamini–Hochberg-adjusted P* values. Necroptosis-related genes were retrieved from the *Mus musculus* necroptosis pathway in the Kyoto Encyclopedia of Genes and Genomes database (KEGG; mmu04217) (31). After gene-symbol harmonization and removal of duplicate entries, 176 unique necroptosis-related genes (NRGs) were retained for subsequent analysis. The complete gene list is provided in Supplementary Table S1. NRDEGs were identified by intersecting DEGs with this predefined NRG set.

### 2.3 Functional enrichment analysis and protein–protein interaction network construction

To investigate the biological significance of DEGs and NRDEGs, Gene Ontology (GO) and Kyoto Encyclopedia of Genes and Genomes (KEGG) enrichment analyses were performed using the “clusterProfiler” package. Biological processes (BP), cellular components (CC), molecular functions (MF), and signaling pathways were analyzed. Enriched terms with adjusted *p* < 0.05 were considered statistically significant.

Protein–protein interaction (PPI) networks were constructed using the STRING database, and only interactions with a combined interaction score ≥ 0.700 (high confidence) were retained. GeneMANIA was used for complementary functional association analysis with automatic network weighting, integrating evidence from predicted interactions, co-expression, disease associations, co-localization, and shared protein domains.

### 2.4 Machine learning-based prioritization of candidate genes

To prioritize candidate genes for exploratory analysis, least absolute shrinkage and selection operator (LASSO) regression was performed using the “glmnet” package. 10-fold cross-validation was applied to determine the penalty parameter used for feature selection.

Random forest (RF) analysis was subsequently conducted using the “randomForest” package. Genes retained by both LASSO and RF analyses were designated candidate genes for further characterization rather than independently validated biomarkers.

### 2.5 Animals and bile duct ligation model

Male C57BL/6 J mice (8 weeks old, 20–22 g) were obtained from Beijing HFK Biotechnology Co., Ltd. (Beijing, China). Animals were housed under specific pathogen-free conditions with a 12-h light/dark cycle and free access to standard chow and water.

Mice were randomly assigned to either the sham-operated (SH, *n* = 6) or bile duct ligation (BDL, *n* = 6) group. Mice were anesthetized with isoflurane delivered in oxygen using an inhalation anesthesia system. Anesthesia was induced with 3% isoflurane and maintained with 1.5% isoflurane during surgery. Adequate anesthetic depth was confirmed by the absence of the pedal withdrawal reflex. A midline laparotomy was performed to expose the common bile duct. The bile duct was doubly ligated with 6–0 silk sutures to induce obstructive cholestasis. Sham-operated mice underwent identical procedures without bile duct ligation.

Twenty-four hours after surgery, mice were deeply anesthetized with isoflurane delivered in oxygen using an inhalation anesthesia system (3% for induction and 1.5% for maintenance). Adequate anesthetic depth was confirmed by the absence of the pedal withdrawal reflex, after which terminal blood samples were collected by cardiac puncture for biochemical analyses. The mice were then euthanized by gradually introducing carbon dioxide into the euthanasia chamber at a displacement rate of 30% of the chamber volume per minute. Carbon dioxide flow was maintained for 1 min after cessation of respiration, and death was confirmed by cervical dislocation. Liver tissues were subsequently harvested for histological and molecular analyses. The 24-h time point was selected because it was the common post-BDL sampling interval in both GEO discovery datasets. This design aligned the experimental validation with the transcriptomic analyses and enabled assessment of early molecular responses before advanced fibrotic remodeling. Thus, the experimental findings primarily represent the acute phase of BDL-induced cholestatic liver injury.

All animal experiments were approved by the Animal Ethics Committee of Shengjing Hospital, China Medical University (Approval No. 2025PS1595K) and were conducted in accordance with the National Institutes of Health Guide for the Care and Use of Laboratory Animals.

### 2.6 Assessment of liver injury and cholestasis

Serum alanine aminotransferase (ALT), aspartate aminotransferase (AST), total bilirubin (TBIL), direct bilirubin (DBIL), and total bile acid (TBA) concentrations were measured using commercial assay kits (Nanjing Jiancheng Bioengineering Institute, Nanjing, China) according to the manufacturer’s instructions.

### 2.7 Histopathological examination

Liver tissues were fixed in 4% paraformaldehyde, embedded in paraffin, cut into 4-μm sections, and stained with hematoxylin and eosin (H&E) for histopathological evaluation.

### 2.8 Cell death assessment

Terminal deoxynucleotidyl transferase dUTP nick-end labeling (TUNEL) staining was performed using a commercial apoptosis detection kit (Beyotime Biotechnology, Shanghai, China) according to the manufacturer’s instructions. TUNEL positivity was used as an indicator of DNA fragmentation and tissue injury; this assay does not distinguish apoptosis from necroptosis or other forms of cell death.

### 2.9 Quantitative real-time PCR

Total RNA was extracted from liver tissues using TRIzol reagent (Invitrogen, USA). RNA was reverse-transcribed into cDNA using HiScript III SuperMix (Vazyme, China). Quantitative PCR was performed using ChamQ Universal SYBR qPCR Master Mix (Vazyme, China). *Gapdh* served as the internal reference gene. Relative mRNA expression levels were calculated using the 2^−ΔΔCt^ method. Primer sequences are listed in Supplementary Table S2.

### 2.10 Western blot analysis

Total protein was extracted from liver tissues using RIPA lysis buffer supplemented with protease and phosphatase inhibitors (Beyotime Biotechnology, Shanghai, China). Protein concentrations were determined using a bicinchoninic acid (BCA) protein assay kit (Beyotime Biotechnology).

Equal amounts of protein (20–30 μg) were separated by 10% sodium dodecyl sulfate–polyacrylamide gel electrophoresis (SDS–PAGE) and transferred onto polyvinylidene difluoride (PVDF) membranes (Millipore, Billerica, MA, United States). Membranes were blocked with 5% non-fat milk for 1 h at room temperature and incubated overnight at 4 °C with primary antibodies against glutamine synthetase (GLUL), receptor-interacting protein kinase 3 (RIP3), phospho-RIP3 (p-RIP3), mixed lineage kinase domain-like protein (MLKL), phospho-mixed lineage kinase domain-like protein (p-MLKL), β-tubulin, and GAPDH (Proteintech, Wuhan, China).

After washing with Tris-buffered saline containing Tween 20 (TBST), membranes were incubated with horseradish peroxidase-conjugated secondary antibodies (Proteintech, Wuhan, China) for 1 h at room temperature. Protein bands were visualized using enhanced chemiluminescence (ECL) reagents (Beyotime Biotechnology, Shanghai, China) and quantified using ImageJ software (National Institutes of Health, Bethesda, MD, United States). GLUL abundance was normalized to β-tubulin, whereas RIP3, p-RIP3, MLKL, and p-MLKL abundance was normalized to GAPDH. For each biological replicate, phosphorylation-to-total-protein ratios were calculated as (p-RIP3/GAPDH)/(RIP3/GAPDH) and (p-MLKL/GAPDH)/(MLKL/GAPDH), respectively.

### 2.11 Statistical analysis

Statistical analyses and graph generation were performed using GraphPad Prism (version 10.0) and R software (version 4.2.0). Quantitative data are presented as mean ± standard deviation (SD) unless otherwise indicated. The expression levels of *Sqstm1*, *Ifngr1*, and *Glul* in the integrated GEO dataset were compared between the BDL and SH groups using unpaired two-sided Welch’s *t*-tests. For experimental data, normality was assessed before statistical testing. Comparisons between two independent groups were performed using unpaired two-sided Student’s t-tests for normally distributed data or Mann–Whitney U tests for non-normally distributed data. The p-RIP3/RIP3 and p-MLKL/MLKL ratios satisfied the normality assumption and were analyzed using unpaired two-sided Student’s *t*-tests. Correlation analyses were conducted using Spearman’s rank correlation coefficient. A two-sided *p* < 0.05 was considered statistically significant.

## 3 Results

### 3.1 Identification of DEGs

We aggregated and integrated the 24-h datasets from GSE166867 and GSE29776 using the R packages “sva” and “limma” for probe annotation, normalization, and reduction of batch-related variation. Principal component analysis and expression-distribution boxplots indicated reduced between-dataset variation after preprocessing (Figures 1A–D). The combined GEO dataset included eight BDL liver samples and six SH liver samples. A total of 813 DEGs were identified, including 461 upregulated and 352 downregulated genes. These DEGs were visualized using a volcano plot (Figure 1E). To visualize the most pronounced transcriptional changes, DEGs meeting *Benjamini–Hochberg-adjusted p* < 0.05 and |log2FC| > 0.5 were ranked by log2FC, and the 20 most upregulated and 20 most downregulated DEGs were displayed in the heatmap (Figure 1F). Values were standardized by gene and are shown as *z*-scores.

**Figure 1:**
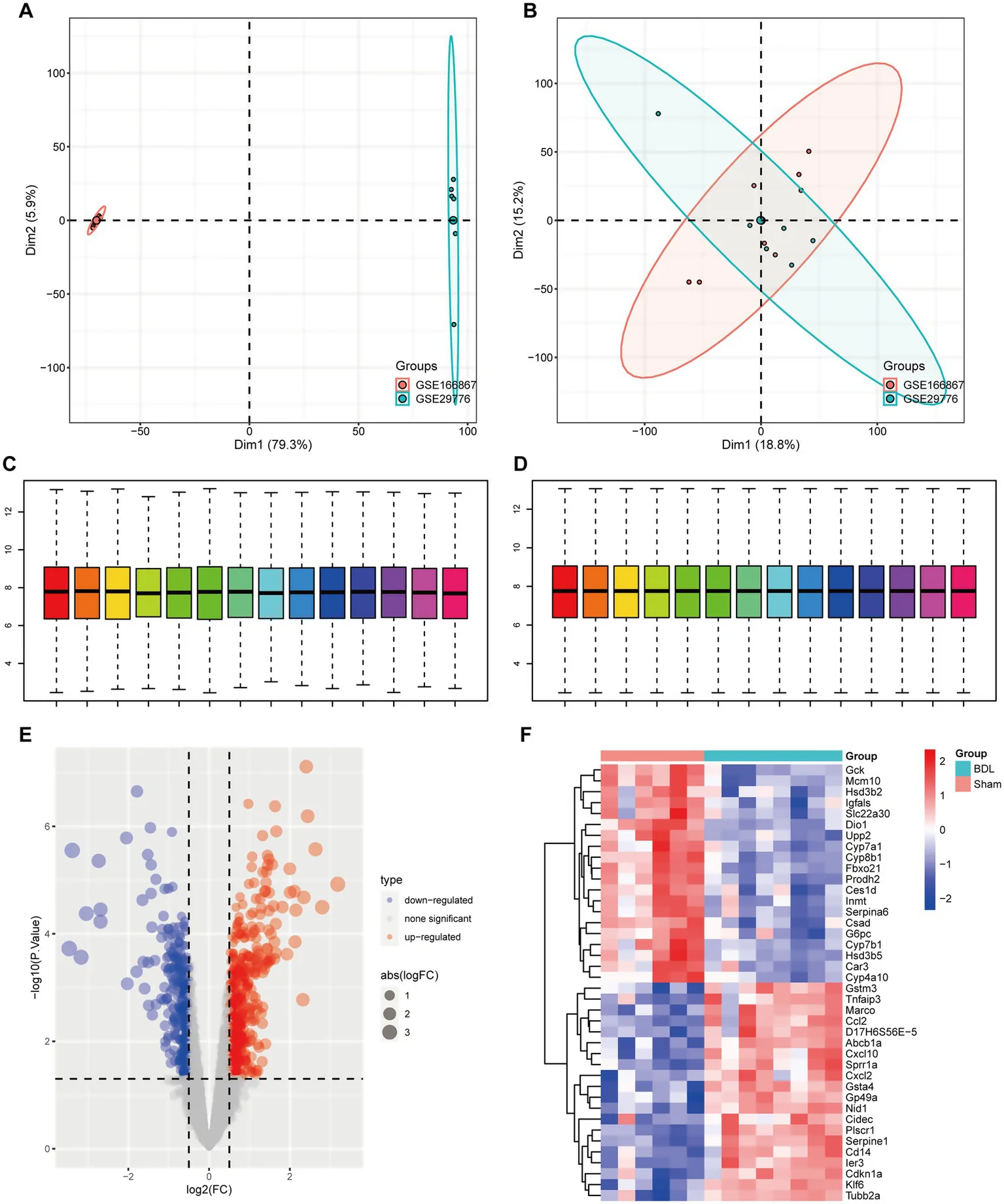
Identification of differentially expressed genes (DEGs) in cholestatic liver injury. **(A,B)** Principal component analysis (PCA) plots showing sample distributions before **(A)** and after **(B)** batch correction. **(C,D)** Boxplots illustrating gene-expression distributions before **(C)** and after **(D)** batch correction. **(E)** Volcano plot of the transcriptomic dataset. DEGs were defined by Benjamini–Hochberg-adjusted *p* < 0.05 and |log2FC| > 0.5. **(F)** Heatmap showing the top 20 upregulated and top 20 downregulated DEGs ranked by log2FC. Colors represent row-wise z-scores of normalized gene-expression values across samples.

### 3.2 Functional enrichment analysis of DEGs

To investigate the biological processes underlying cholestatic liver injury, GO and KEGG enrichment analyses were performed on the identified DEGs. GO analysis revealed significant enrichment in cytokine-mediated signaling pathways, leukocyte migration, steroid metabolic processes, purine-containing compound metabolic processes, and organic acid biosynthetic processes (Figure 2A). Several enriched biological processes were related to nutrient metabolism and metabolic homeostasis, suggesting that early cholestatic injury is accompanied by transcriptional alterations in pathways involved in metabolic regulation.

**Figure 2:**
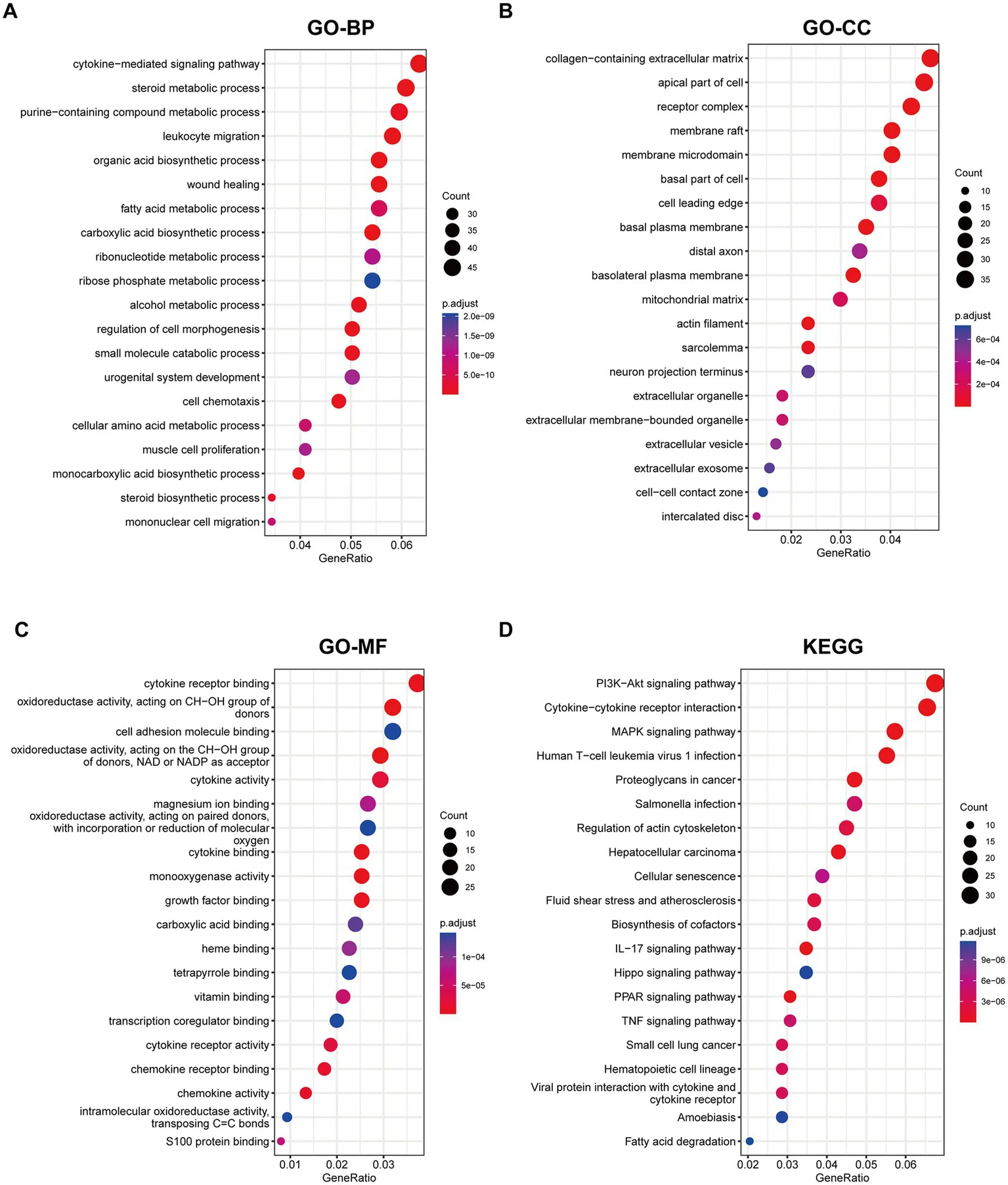
Functional enrichment analysis of DEGs in early cholestatic liver injury. **(A)** Gene Ontology (GO) biological process (BP) enrichment analysis. **(B)** GO cellular component (CC) enrichment analysis. **(C)** GO molecular function (MF) enrichment analysis. **(D)** Kyoto Encyclopedia of Genes and Genomes (KEGG) pathway enrichment analysis. The enriched terms include pathways related to metabolic regulation, inflammatory signaling, and cellular stress responses. GO, Gene Ontology; KEGG, Kyoto Encyclopedia of Genes and Genomes.

Cellular component analysis demonstrated enrichment in collagen-containing extracellular matrix, receptor complexes, membrane rafts, and membrane microdomains (Figure 2B). Molecular function analysis revealed significant enrichment in cytokine receptor binding, cytokine binding, cell adhesion molecule binding, and oxidoreductase activity (Figure 2C).

KEGG enrichment analysis identified significant enrichment in the PI3K–Akt signaling pathway, cytokine–cytokine receptor interaction, MAPK signaling pathway, and other inflammatory signaling pathways (Figure 2D). Collectively, these enrichment results suggest coordinated transcriptional alterations in pathways related to metabolic regulation, inflammatory signaling, and cellular stress responses during early cholestatic injury.

### 3.3 Identification and functional enrichment analysis of NRDEGs

Eight necroptosis-related differentially expressed genes (NRDEGs) were identified, including *Tnfaip3*, *Sqstm1*, *Chmp2b*, *Ifngr2*, *Slc25a4*, *Ifngr1*, *Camk2d*, and *Glul*. Seven genes were significantly upregulated, whereas *Glul* was significantly downregulated in BDL liver tissues (Figures 3A,B).

**Figure 3:**
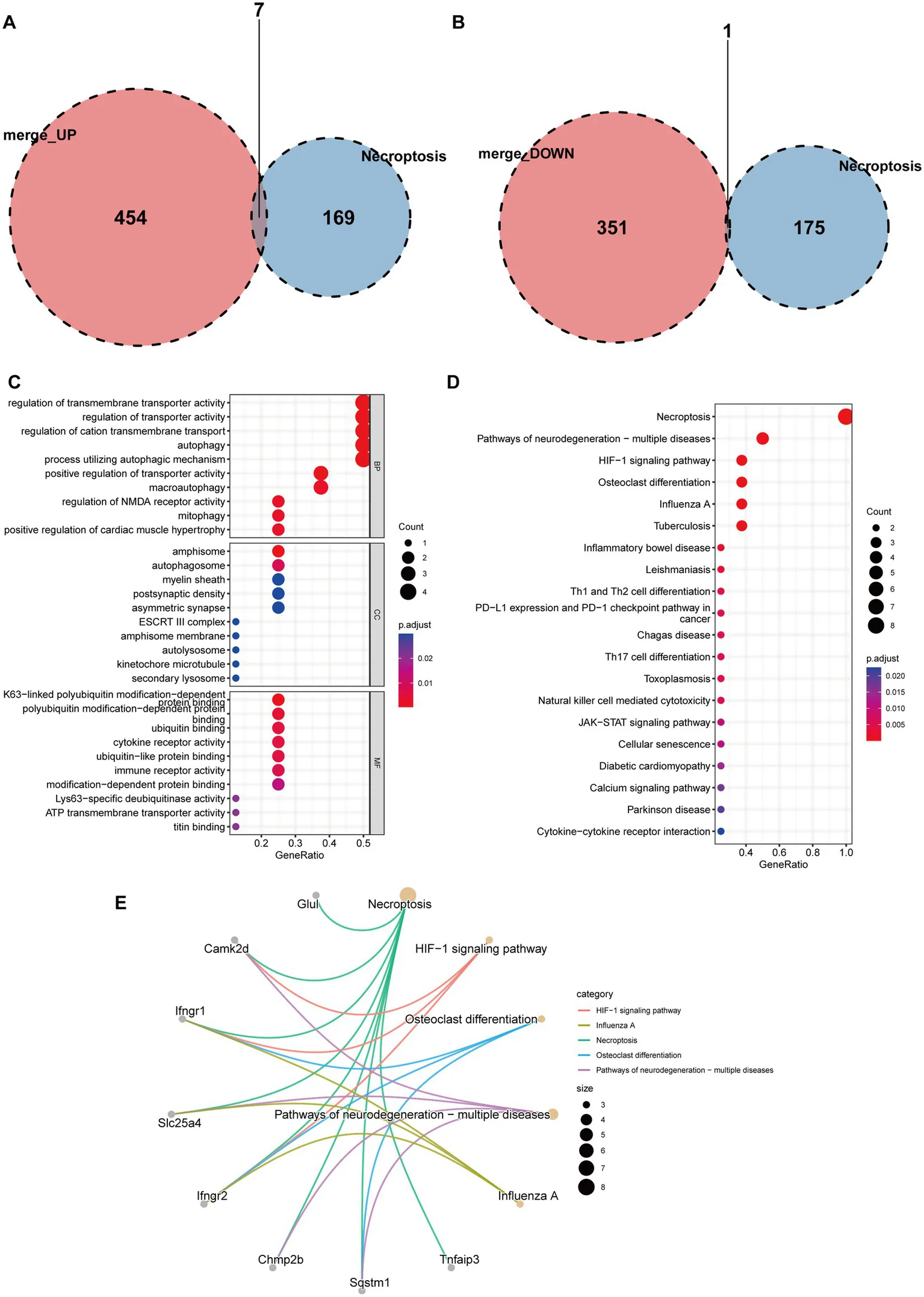
Identification and functional enrichment analysis of necroptosis-related differentially expressed genes (NRDEGs). **(A)** Venn diagram showing the overlap between necroptosis-related genes (NRGs) and upregulated DEGs. **(B)** Venn diagram showing the overlap between NRGs and downregulated DEGs. **(C)** GO enrichment analysis of NRDEGs. **(D)** KEGG pathway enrichment analysis of NRDEGs. **(E)** Relationships between the top five enriched KEGG pathways and NRDEGs.

Functional enrichment analysis revealed that NRDEGs were involved not only in necroptosis-related pathways but also in biological processes associated with metabolic regulation. GO analysis demonstrated significant enrichment in the regulation of transmembrane transporter activity, cation transport, and autophagy-related processes (Figure 3C). KEGG analysis further identified significant enrichment in necroptosis, HIF-1 signaling, and inflammation-related pathways (Figure 3D). The relationships between the top enriched pathways and NRDEGs are illustrated in Figure 3E.

### 3.4 Expression patterns of NRDEGs

The expression patterns of the eight NRDEGs were further visualized using a volcano plot and individual boxplots ((Figures 4A–C)). A heatmap of 21 genes, including the eight NRDEGs, is shown in (Figure 4B).

**Figure 4:**
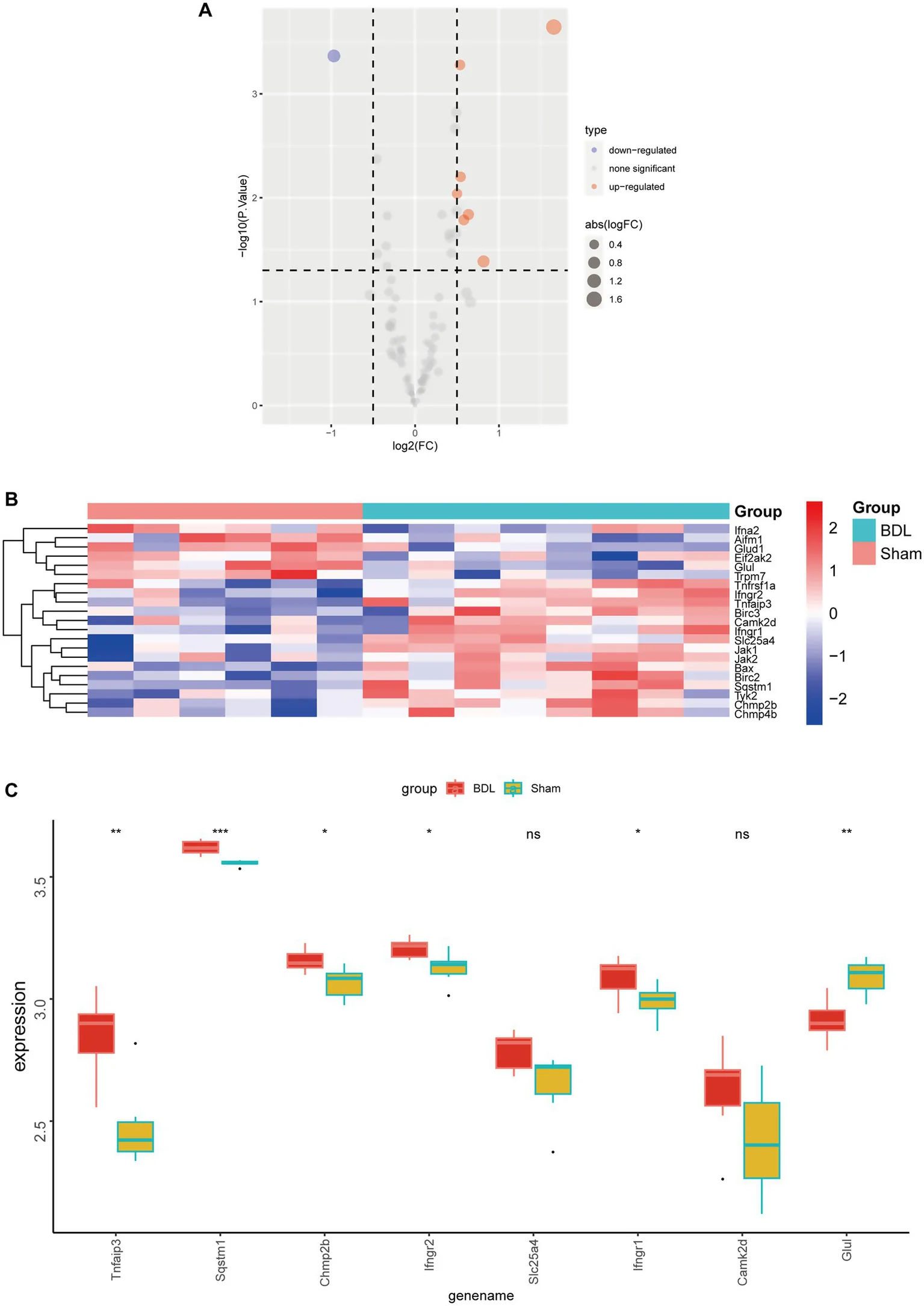
Differential expression patterns of NRDEGs in cholestatic liver injury. **(A)** Volcano plot of NRDEGs. **(B)** Heatmap showing expression patterns of 21 genes, including all eight NRDEGs, in BDL and SH groups. **(C)** Boxplots illustrating expression levels of individual NRDEGs. Significance labels in **(C)** are from two-group t-tests and may differ from limma results. ns, *p* > 0.05; *p < 0.05; **p < 0.01; ***p < 0.001.

### 3.5 PPI analysis

The STRING PPI network comprised eight nodes and four edges, including three isolated nodes (Figure 5A). GeneMANIA identified functionally associated genes and highlighted processes related to transporter regulation and cellular metabolism (Figure 5B). These network-level associations are exploratory.

**Figure 5:**
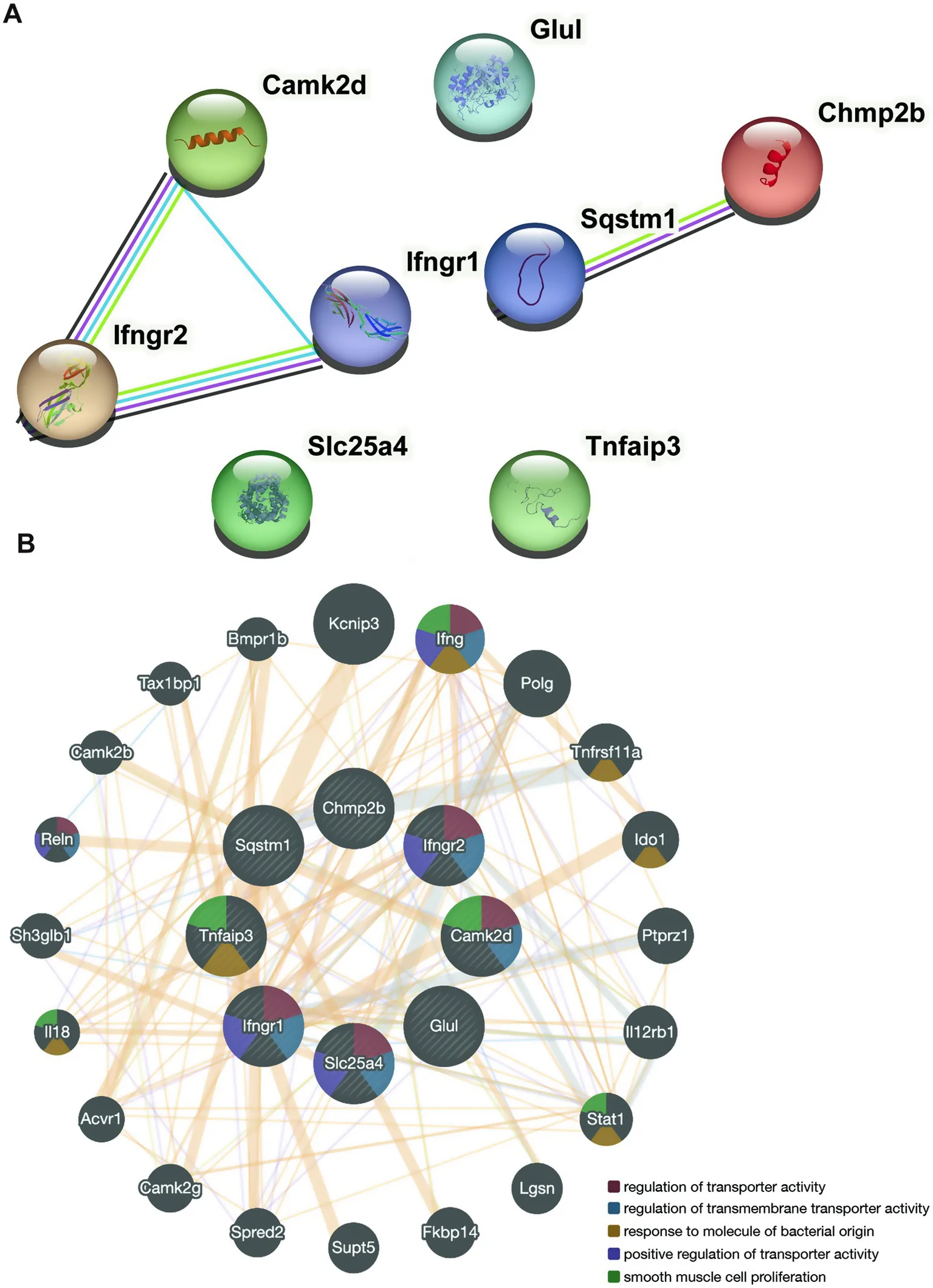
Protein–protein interaction and functional association network analyses of NRDEGs. **(A)** STRING-based PPI network constructed using a combined interaction score ≥ 0.700 (high confidence). **(B)** GeneMANIA functional association network generated using automatic network weighting and multiple evidence sources.

### 3.6 Exploratory prioritization of candidate NRDEGs

To prioritize NRDEGs for further exploratory characterization, LASSO regression and RF analysis were applied. LASSO retained *Sqstm1*, *Chmp2b*, *Slc25a4*, *Ifngr1*, and *Glul* (Figure 6A), whereas RF ranked *Sqstm1*, *Tnfaip3*, *Ifngr2*, *Glul*, and *Ifngr1* among the five most important genes (Figure 6B).

**Figure 6:**
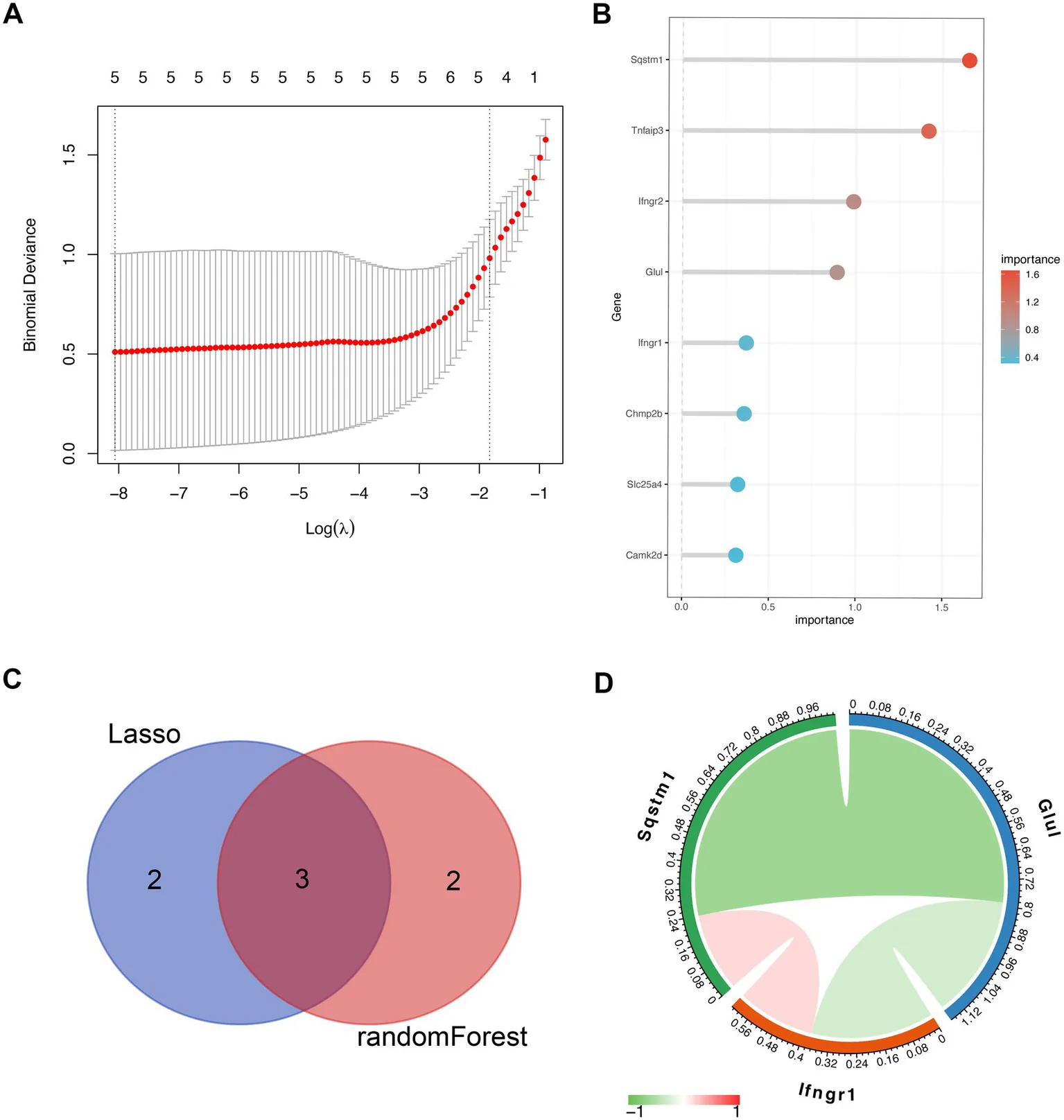
Exploratory machine-learning prioritization and correlation analysis of candidate NRDEGs. **(A)** Feature selection using least absolute shrinkage and selection operator (LASSO) regression. **(B)** Feature ranking using random forest (RF) analysis. **(C)** Venn diagram showing Sqstm1, Ifngr1, and Glul retained by both algorithms. **(D)** Correlation analysis among Sqstm1, Ifngr1, and Glul using Spearman’s rank correlation coefficient.

The overlap between the two feature-prioritization approaches comprised *Sqstm1*, *Ifngr1*, and *Glul* (Figure 6C). These genes were retained as exploratory candidates associated with metabolic and inflammatory responses in cholestatic liver injury. *Glul* was the only downregulated candidate and was therefore prioritized for further characterization. Because both algorithms were applied to the same small discovery dataset, these selections require independent external validation.

### 3.7 Correlation and expression analysis of candidate NRDEGs

Correlation analysis showed that Sqstm1 was negatively correlated with Glul and positively correlated with Ifngr1, whereas Glul was negatively correlated with Ifngr1 (Figure 6D). Expression analysis showed increased Sqstm1 and Ifngr1 expression and decreased Glul expression in BDL liver tissues compared with SH controls (Figures 7A–C). Exploratory ROC curves are presented in Supplementary Figure S1. Because candidate prioritization and ROC assessment were performed using the same 14 samples, these results are descriptive and should not be interpreted as independently validated discriminatory performance.

**Figure 7:**
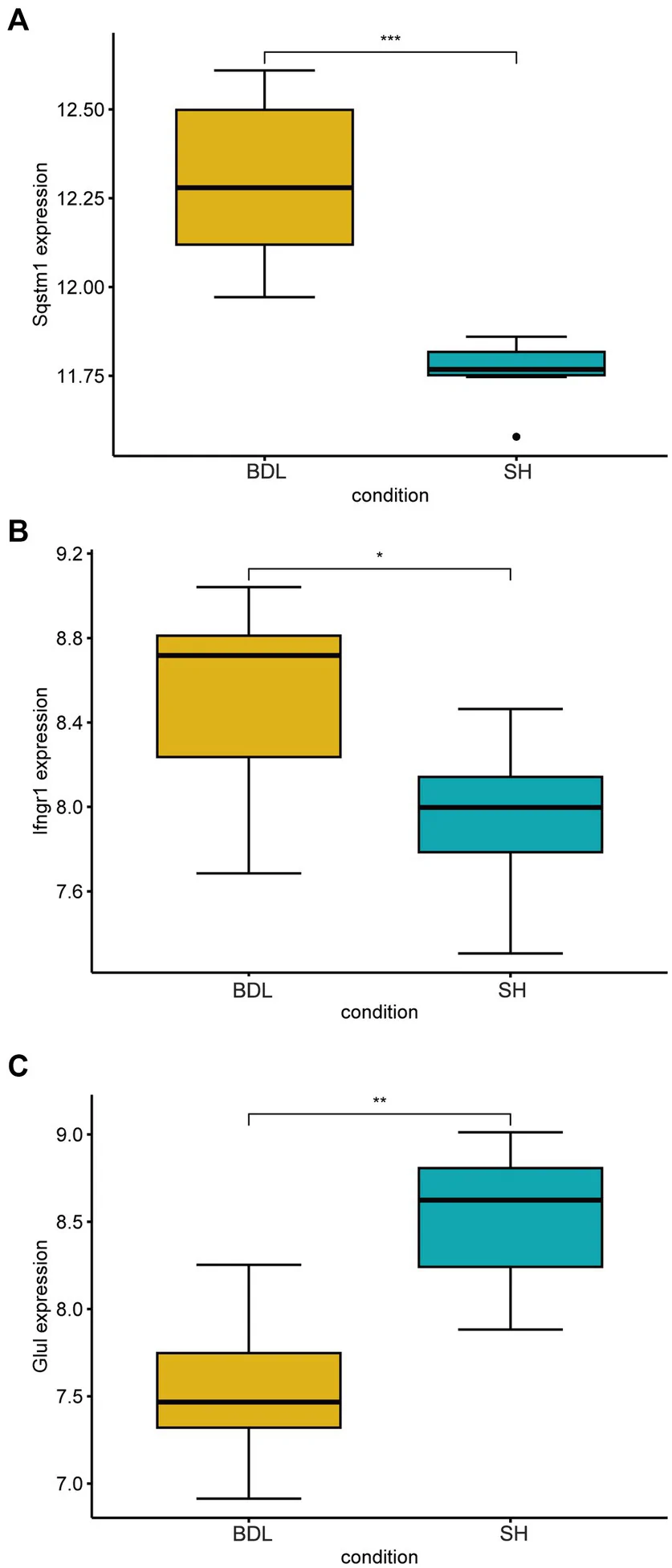
Differential expression of candidate genes in liver tissues from BDL and sham-operated mice. **(A)** *Sqstm1*, **(B)** *Ifngr1*, and **(C)** *Glul* expression levels are presented as boxplots. ns, *p* > 0.05; *p < 0.05; **p < 0.01; ***p < 0.001.

### 3.8 Biochemical parameters and histological changes in the BDL model

The BDL group exhibited significantly higher serum AST, ALT, TBA, TBIL, and DBIL levels than the SH group (Figures 8A–E). H&E staining showed marked hepatocellular injury, parenchymal disruption, loss of normal lobular architecture, and inflammatory-cell accumulation around portal areas in the BDL group (Figure 9A). These biochemical and histopathological changes supported the successful establishment of the early cholestatic liver injury model.

**Figure 8:**
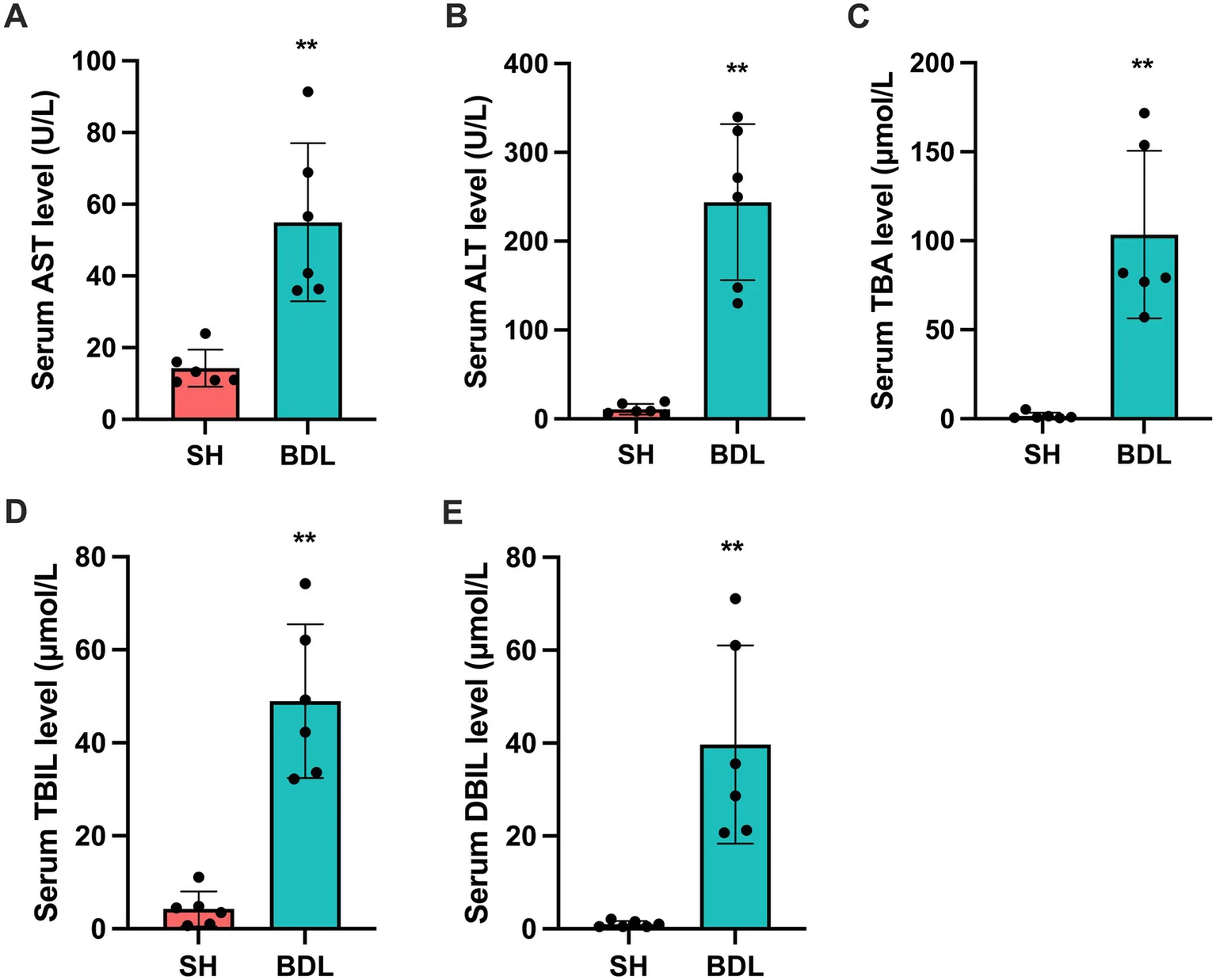
Biochemical evidence of liver injury and cholestasis following bile duct ligation. Serum levels of **(A)** AST, **(B)** ALT, **(C)** TBA, **(D)** TBIL, and **(E)** DBIL. Data are presented as mean ± SD (*n* = 6 per group). **p < 0.01.

**Figure 9:**
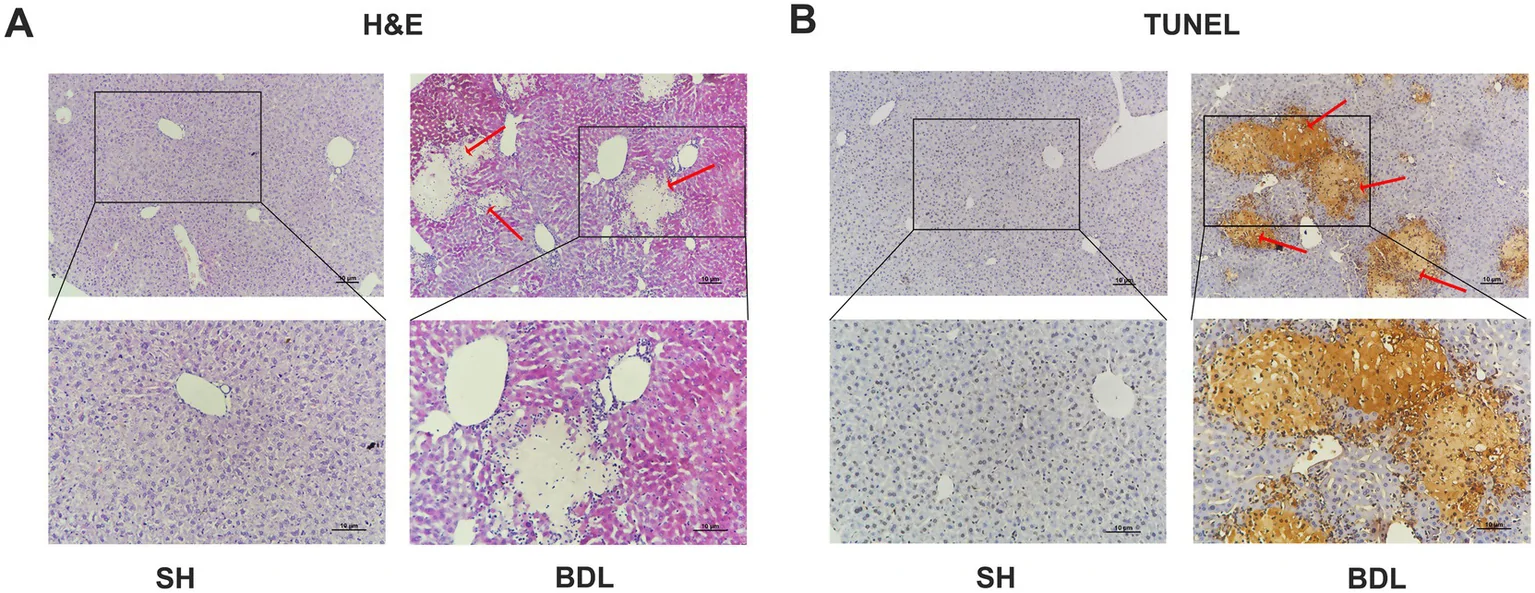
Histopathological alterations and DNA fragmentation in cholestatic liver injury. **(A)** Representative hematoxylin and eosin (H&E)-stained liver sections from the SH and BDL groups. Red arrows indicate representative areas of hepatocellular injury and inflammatory-cell accumulation. **(B)** Representative TUNEL-stained liver sections from the SH and BDL groups. Red arrows indicate representative TUNEL-positive areas. Boxed regions are shown at higher magnification below. TUNEL positivity reflects DNA fragmentation and does not distinguish apoptosis from necroptosis or other forms of cell death. Scale bars, 10 μm.

### 3.9 Experimental assessment of candidate genes and RIP3/MLKL-pathway proteins

TUNEL staining showed increased TUNEL positivity in BDL liver sections relative to SH controls, indicating enhanced DNA fragmentation during early cholestatic injury (Figure 9B). Because TUNEL does not identify a specific mode of cell death, this observation was not interpreted as direct evidence of necroptosis.

RT-qPCR analysis demonstrated significantly increased mRNA expression of Ripk1, Ripk3, Mlkl, Sqstm1, and Ifngr1, whereas Glul mRNA expression was significantly decreased in BDL liver tissues (Figures 10A–F).

**Figure 10:**
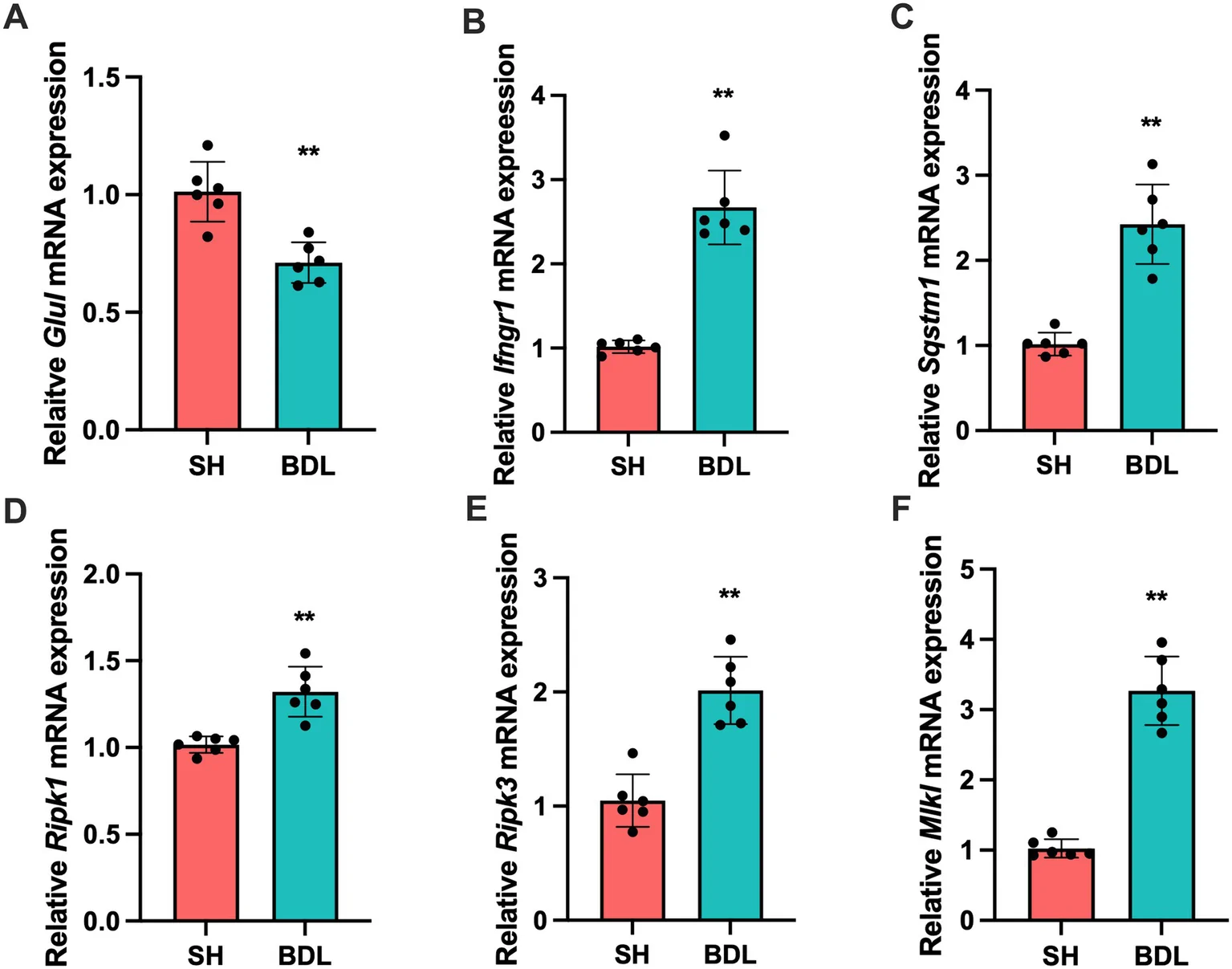
Experimental assessment of candidate genes and necroptosis-associated markers by RT-qPCR. Relative mRNA expression levels of **(A)** *Glul*, **(B)** *Ifngr1*, **(C)** *Sqstm1*, **(D)** *Ripk1*, **(E)** *Ripk3*, and **(F)** *Mlkl* in SH and BDL liver tissues. *Gapdh* served as the internal control. Data are presented as mean ± SD (*n* = 6 per group). **p < 0.01.

Western blot analysis showed reduced GLUL protein abundance and increased GAPDH-normalized RIP3, p-RIP3, MLKL, and p-MLKL abundance in BDL liver tissues (Figures 11A–F). Importantly, both the p-RIP3/RIP3 and p-MLKL/MLKL ratios were significantly increased in the BDL group compared with the SH group (*p* < 0.05; Figures 11G,H), indicating enhanced phosphorylation of RIP3 and MLKL relative to the corresponding total-protein levels. These findings support enhanced RIP3/MLKL-associated signaling during early BDL-induced cholestatic liver injury.

**Figure 11:**
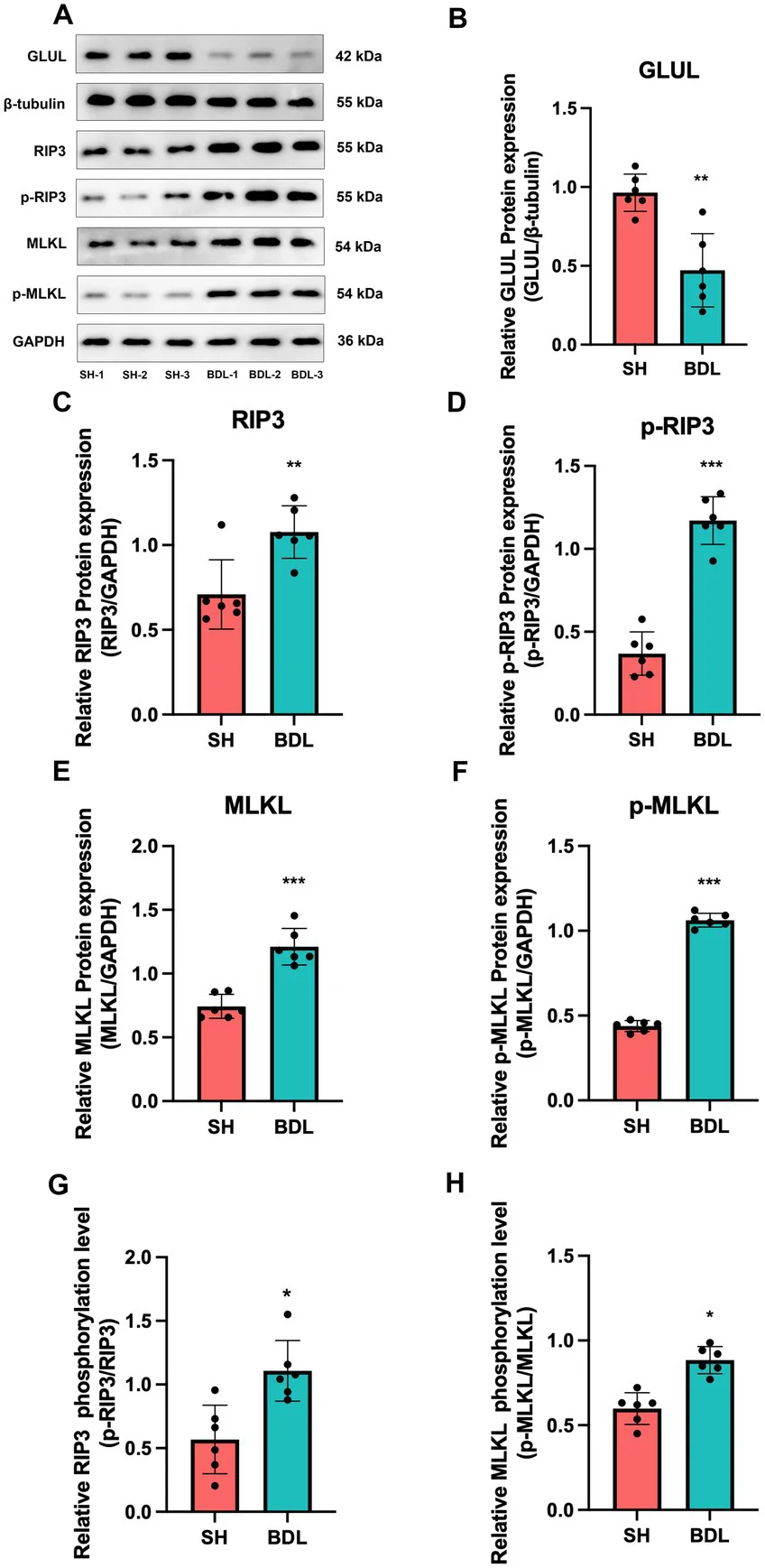
Western blot assessment of GLUL and RIP3/MLKL-pathway proteins. **(A)** Representative immunoblots of GLUL, β-tubulin, RIP3, p-RIP3, MLKL, p-MLKL, and GAPDH. Three representative samples from each group are shown. Quantification of **(B)** GLUL normalized to β-tubulin and **(C)** RIP3, **(D)** p-RIP3, **(E)** MLKL, and **(F)** p-MLKL normalized to GAPDH. **(G)** p-RIP3/RIP3 ratio. **(H)** p-MLKL/MLKL ratio. Ratios were calculated for each biological replicate from the corresponding GAPDH-normalized values. Data are presented as mean ± SD (*n* = 6 per group). *p < 0.05; **p < 0.01; ***p < 0.001.

## 4 Discussion

Obstructive cholestasis is increasingly recognized as a complex disorder involving disrupted bile acid homeostasis, hepatocellular injury, inflammation, and transcriptional alterations in metabolic pathways. In the present study, integrated transcriptomic analyses identified eight NRDEGs and three exploratory candidate genes (*Sqstm1*, *Ifngr1*, and *Glul*). Experimental assessment demonstrated concurrent GLUL downregulation, increased GAPDH-normalized RIP3, p-RIP3, MLKL, and p-MLKL abundance, and significant increases in both p-RIP3/RIP3 and p-MLKL/MLKL during early BDL-induced injury.

### 4.1 Cholestatic liver injury is accompanied by metabolic pathway alterations

Bile acids are increasingly recognized as metabolic signaling molecules that regulate glucose, lipid, and amino acid metabolism through FXR and TGR5-mediated pathways. Consistent with this concept, our enrichment analyses identified transcriptional alterations in steroid metabolic processes, organic acid biosynthetic processes, and transmembrane transporter activity. The marked increase in serum TBA confirmed systemic bile acid accumulation and successful induction of cholestasis. Collectively, these findings suggest that early cholestatic injury is accompanied by alterations in pathways related to metabolic regulation; they do not directly demonstrate impairment of glutamine metabolism.

### 4.2 GLUL downregulation as a candidate molecular feature

Among the eight NRDEGs identified in the integrated transcriptomic analysis, Glul was the only downregulated gene. Glul encodes glutamine synthetase (GLUL), a key enzyme involved in glutamine biosynthesis, nitrogen metabolism, and hepatic metabolic homeostasis. GLUL is predominantly expressed in pericentral hepatocytes and plays a critical role in ammonia detoxification and glutamine synthesis in the liver (16). Therefore, reduced Glul expression may be relevant to hepatic nitrogen homeostasis during early cholestatic injury, although its functional consequences remain unknown.

Previous studies have linked alterations in glutamine–glutamate and nitrogen metabolism to metabolic dysfunction and clinical outcomes, although the prognostic relevance of individual metabolites remains context dependent (32, 33). Altered glutamine metabolism has also been implicated in liver fibrosis and hepatocellular carcinoma progression (17, 34). This biological context provides a rationale for further investigating the metabolic implications of reduced Glul expression during cholestatic injury.

Consistent with the transcriptomic finding, reduced *Glul* mRNA expression was reproduced by RT-qPCR in the BDL model, and western blotting showed reduced hepatic GLUL protein abundance. These concordant observations support *Glul*/GLUL downregulation as a reproducible feature of early cholestatic injury.

### 4.3 GLUL downregulation accompanies changes in RIP3/MLKL-pathway protein abundance

Necroptosis has emerged as an important mechanism of regulated cell death in liver diseases (23, 24). Consistent with previous reports, we observed increased *Ripk1*, *Ripk3*, and *Mlkl* mRNA expression in BDL liver tissues. Western blotting also showed increased GAPDH-normalized RIP3, p-RIP3, MLKL, and p-MLKL abundance. Because total RIP3 and total MLKL abundance also increased, we further normalized each phosphorylated protein to its corresponding total protein. Both p-RIP3/RIP3 and p-MLKL/MLKL were significantly increased in the BDL group, indicating that the increases in phosphorylated RIP3 and MLKL were not attributable solely to higher total protein abundance. These findings support enhanced RIP3/MLKL-associated signaling. Nevertheless, increased phosphorylation alone does not establish complete necroptotic flux or pathway dependence.

Emerging evidence indicates that regulated cell-death pathways are influenced by cellular metabolic status (18, 24). In the present study, reduced GLUL expression occurred concurrently with altered RIP3/MLKL-pathway protein abundance and higher p-RIP3/RIP3 and p-MLKL/MLKL ratios. Whether these alterations are temporally or functionally linked remains to be determined. Reduced GLUL expression could instead be secondary to cholestatic injury, including bile acid-mediated hepatocellular injury, inflammatory stress, or loss of the pericentral hepatocyte phenotype, rather than a cause of RIP3/MLKL-pathway changes.

### 4.4 Nutritional implications and potential therapeutic perspectives

The findings suggest that glutamine-related metabolic regulation may be relevant to hepatic homeostasis during cholestasis. However, the current data do not support specific nutritional interventions. Future studies should combine hepatocyte-specific *Glul* knockout or knockdown with rescue or overexpression experiments and assess GLUL activity, glutamine and nitrogen-related metabolites, ammonia detoxification, redox homeostasis, liver injury, and RIP3/MLKL-pathway phosphorylation ratios before glutamine-targeted interventions are evaluated.

During chronic cholestasis, persistent GLUL downregulation could theoretically compromise ammonia detoxification, glutamine availability, and redox homeostasis. Sustained changes in RIP3/MLKL-pathway protein abundance and MLKL phosphorylation could contribute to damage-associated molecular pattern release, ductular reaction, hepatic stellate-cell activation, and fibrotic remodeling. These possibilities were not directly examined and require longitudinal and mechanistic validation.

Because the 24-h model captures only acute injury, longitudinal analyses are needed to determine whether GLUL downregulation precedes, parallels, or follows changes in RIP3/MLKL-pathway protein abundance and fibrotic remodeling. Studies at 1, 3, 7, 14, and 28 days after BDL may help distinguish a causal metabolic alteration from a secondary consequence of hepatocellular injury. Together, these concurrent observations and the remaining mechanistic uncertainties are summarized in the hypothesis-generating framework shown in Figure 12.

**Figure 12:**
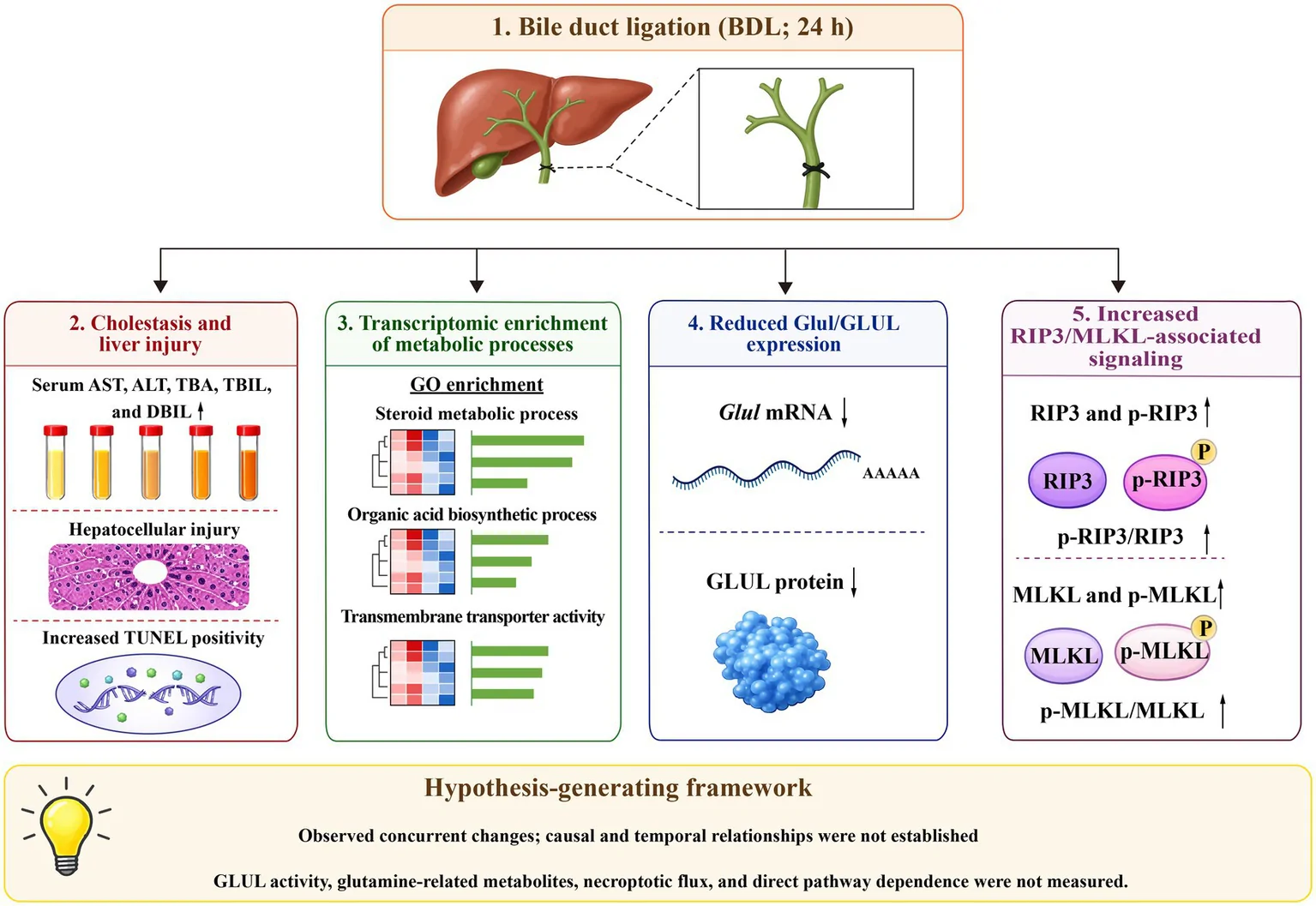
Hypothesis-generating framework summarizing concurrent changes observed during early cholestatic liver injury. Bile duct ligation was accompanied by biochemical and histological liver injury, transcriptomic enrichment of metabolic processes, reduced Glul mRNA expression and GLUL protein abundance, increased GAPDH-normalized RIP3, p-RIP3, MLKL, and p-MLKL abundance, and significant increases in both p-RIP3/RIP3 and p-MLKL/MLKL. Solid arrows indicate concurrent observations in the 24-h BDL model and do not imply causal or temporal relationships. GLUL activity, glutamine-related metabolites, necroptotic flux, and direct pathway dependence were not measured.

### 4.5 Limitations

Several limitations should be acknowledged. The public datasets and murine cohort were small, and the features selected by the machine-learning approaches lacked external validation. The within-dataset ROC analysis is presented only in the Supplementary material and is not interpreted as evidence of validated discriminatory performance. The single 24-h time point limits inference regarding later disease, and the absence of serum alkaline phosphatase (ALP) measurement limits the biochemical characterization of cholestasis. Although both p-RIP3/RIP3 and p-MLKL/MLKL were significantly increased, without pathway inhibition, MLKL oligomerization or membrane-translocation assays, or direct assessment of membrane-disruptive activity, these protein measurements do not establish pathway-dependent necroptotic flux. Without Glul manipulation, causality cannot be established. Glutamine-related metabolites and GLUL enzymatic activity were not measured, and TUNEL does not specifically identify necroptosis. Future studies should address these limitations through longitudinal, mechanistic, and metabolic experiments, with validation in human samples.

## 5 Conclusion

Through integrated transcriptomic analyses and experimental validation, this study identified reduced hepatic *Glul* mRNA expression and GLUL protein abundance as reproducible features of early BDL-induced cholestatic liver injury. GLUL downregulation occurred concurrently with increased GAPDH-normalized RIP3, p-RIP3, MLKL, and p-MLKL abundance and significant increases in both p-RIP3/RIP3 and p-MLKL/MLKL. These findings support enhanced RIP3/MLKL-associated signaling but do not establish pathway-dependent necroptotic flux, a causal relationship with GLUL, or GLUL as a validated therapeutic target.

## Data Availability

Publicly available datasets analyzed in this study can be found in the GEO database under accession numbers GSE29776 and GSE166867.

## References

[1] Lazaridis KN LaRusso NF. The cholangiopathies. Mayo Clin Proc. (2015) 90:791–800. doi: 10.1016/j.mayocp.2015.03.017, 25957621 PMC4533104

[2] Santiago P Scheinberg AR Levy C. Cholestatic liver diseases: new targets, new therapies. Ther Adv Gastroenterol. (2018) 11:1756284818787400. doi: 10.1177/1756284818787400, 30159035 PMC6109852

[3] Trauner M Fuchs CD Halilbasic E Paumgartner G. New therapeutic concepts in bile acid transport and signaling for management of cholestasis. Hepatology. (2017) 65:1393–404. doi: 10.1002/hep.28991, 27997980

[4] Fickert P Wagner M. Biliary bile acids in hepatobiliary injury—what is the link? J Hepatol. (2017) 67:619–31. doi: 10.1016/j.jhep.2017.04.026, 28712691

[5] Li T Chiang JYL. Bile acid signaling in metabolic and inflammatory diseases and drug development. Pharmacol Rev. (2024) 76:1221–53. doi: 10.1124/pharmrev.124.000978, 38977324 PMC11549937

[6] Chiang JYL Ferrell JM. Bile acid receptors FXR and TGR5 signaling in fatty liver diseases and therapy. Am J Physiol Gastrointest Liver Physiol. (2020) 318:G554–73. doi: 10.1152/ajpgi.00223.2019, 31984784 PMC7099488

[7] Simbrunner B Mandorfer M Trauner M Reiberger T. Review article: therapeutic aspects of bile acid signalling in the gut-liver axis. Aliment Pharmacol Ther. (2021) 54:1243–62. doi: 10.1111/apt.16602 PMC9290708 34555862

[8] Hsu CL Schnabl B. The gut-liver axis and gut microbiota in health and liver disease. Nat Rev. Microbiol. (2023) 21:719–33. doi: 10.1038/s41579-023-00904-3, 37316582 PMC10794111

[9] Kim YS Hurley EH Park Y Ko S. Primary sclerosing cholangitis and inflammatory bowel disease: a condition exemplifying the crosstalk of the gut-liver axis. Exp Mol Med. (2023) 55:1380–7. doi: 10.1038/s12276-023-01042-9, 37464092 PMC10394020

[10] Collins SL Stine JG Bisanz JE Okafor CD Patterson AD. Bile acids and the gut microbiota: metabolic interactions and impacts on disease. Nat Rev. Microbiol. (2023) 21:236–47. doi: 10.1038/s41579-022-00805-x, 36253479 PMC12536349

[11] Peiseler M Schwabe R Hampe J Kubes P Heikenwälder M Tacke F. Immune mechanisms linking metabolic injury to inflammation and fibrosis in fatty liver disease: novel insights into cellular communication circuits. J Hepatol. (2022) 77:1136–60. doi: 10.1016/j.jhep.2022.06.012, 35750137

[12] Cruzat V Macedo Rogero M Noel Keane K Curi R Newsholme P. Glutamine: metabolism and immune function, supplementation and clinical translation. Nutrients. (2018) 10:1564. doi: 10.3390/nu10111564, 30360490 PMC6266414

[13] Yoo HC Yu YC Sung Y Han JM. Glutamine reliance in cell metabolism. Exp Mol Med. (2020) 52:1496–516. doi: 10.1038/s12276-020-00504-8, 32943735 PMC8080614

[14] Newsholme P Diniz VLS Dodd GT Cruzat V. Glutamine metabolism and optimal immune and CNS function. Proc Nutr Soc. (2023) 82:22–31. doi: 10.1017/S0029665122002749, 36285520

[15] Xuan DTM Wu CC Wang WJ Hsu HP Ta HDK Anuraga G . Glutamine synthetase regulates the immune microenvironment and cancer development through the inflammatory pathway. Int J Med Sci. (2023) 20:35–49. doi: 10.7150/ijms.75625, 36619229 PMC9812810

[16] Villar VH Allega MF Deshmukh R Ackermann T Nakasone MA Vande Voorde J . Hepatic glutamine synthetase controls N5-methylglutamine in homeostasis and cancer. Nat Chem Biol. (2023) 19:292–300. doi: 10.1038/s41589-022-01154-9, 36280791 PMC9974483

[17] Yin X Peng J Gu L Liu Y Li X Wu J . Targeting glutamine metabolism in hepatic stellate cells alleviates liver fibrosis. Cell Death Dis. (2022) 13:955. doi: 10.1038/s41419-022-05409-0, 36376267 PMC9663710

[18] Bertheloot D Latz E Franklin BS. Necroptosis, pyroptosis and apoptosis: an intricate game of cell death. Cell Mol Immunol. (2021) 18:1106–21. doi: 10.1038/s41423-020-00630-3, 33785842 PMC8008022

[19] Newton K. RIPK1 and RIPK3: critical regulators of inflammation and cell death. Trends Cell Biol. (2015) 25:347–53. doi: 10.1016/j.tcb.2015.01.001, 25662614

[20] Frank D Vince JE. Pyroptosis versus necroptosis: similarities, differences, and crosstalk. Cell Death Differ. (2019) 26:99–114. doi: 10.1038/s41418-018-0212-6, 30341423 PMC6294779

[21] Chen X Li W Ren J Huang D He WT Song Y . Translocation of mixed lineage kinase domain-like protein to plasma membrane leads to necrotic cell death. Cell Res. (2014) 24:105–21. doi: 10.1038/cr.2013.171, 24366341 PMC3879712

[22] Wang H Sun L Su L Rizo J Liu L Wang LF . Mixed lineage kinase domain-like protein MLKL causes necrotic membrane disruption upon phosphorylation by RIP3. Mol Cell. (2014) 54:133–46. doi: 10.1016/j.molcel.2014.03.003, 24703947

[23] Afonso MB Rodrigues PM Simão AL Ofengeim D Carvalho T Amaral JD . Activation of necroptosis in human and experimental cholestasis. Cell Death Dis. (2016) 7:e2390. doi: 10.1038/cddis.2016.280, 27685634 PMC5059878

[24] Xue C Gu X Li G Bao Z Li L. Mitochondrial mechanisms of necroptosis in liver diseases. Int J Mol Sci. (2020) 22:66. doi: 10.3390/ijms22010066, 33374660 PMC7793526

[25] Chen J Zhang S. The role of inflammation in cholestatic liver injury. J Inflamm Res. (2023) 16:4527–40. doi: 10.2147/JIR.S430730, 37854312 PMC10581020

[26] Guillot A Tacke F. Spatial dimension of macrophage heterogeneity in liver diseases. eGastroenterology. (2023) 1:e000003. doi: 10.1136/egastro-2023-000003, 39944246 PMC11770455

[27] Woolbright BL Jaeschke H. Therapeutic targets for cholestatic liver injury. Expert Opin Ther Targets. (2016) 20:463–75. doi: 10.1517/14728222.2016.1103735, 26479335 PMC4890577

[28] Li M Cai SY Boyer JL. Mechanisms of bile acid mediated inflammation in the liver. Mol Asp Med. (2017) 56:45–53. doi: 10.1016/j.mam.2017.06.001, 28606651 PMC5662014

[29] Yokoda RT Rodriguez EA. Review: pathogenesis of cholestatic liver diseases. World J Hepatol. (2020) 12:423–35. doi: 10.4254/wjh.v12.i8.423, 32952871 PMC7475774

[30] Heymann F Tacke F. Immunology in the liver—from homeostasis to disease. Nat Rev. Gastroenterol Hepatol. (2016) 13:88–110. doi: 10.1038/nrgastro.2015.200, 26758786

[31] Kanehisa M Furumichi M Sato Y Matsuura Y Ishiguro-Watanabe M. KEGG: biological systems database as a model of the real world. Nucleic Acids Res. (2025) 53:D672–7. doi: 10.1093/nar/gkae909, 39417505 PMC11701520

[32] Wunderle C von Arx D Mueller SC Bernasconi L Neyer P Tribolet P . Association of glutamine and glutamate metabolism with mortality among patients at nutritional risk: a secondary analysis of the randomized clinical trial EFFORT. Nutrients. (2024) 16:222. doi: 10.3390/nu16020222, 38257115 PMC10821126

[33] Delgado TC de las Heras J Martínez-Chantar ML. Understanding gut-liver axis nitrogen metabolism in fatty liver disease. Front Endocrinol (Lausanne). (2022) 13:1058101. doi: 10.3389/fendo.2022.1058101, 36589817 PMC9797658

[34] Ye Y Yu B Wang H Yi F. Glutamine metabolic reprogramming in hepatocellular carcinoma. Front Mol Biosci. (2023) 10:1242059. doi: 10.3389/fmolb.2023.1242059, 37635935 PMC10452011

