# Supplementary material for: Reduced hepatic GLUL expression accompanies altered RIP3/MLKL-pathway protein abundance in early cholestatic liver injury following bile duct ligation
Source: Front Nutr. 2026 Oct 2;13:1912372. doi: 10.3389/fnut.2026.1912372 (PMC13672626; doi:10.3389/fnut.2026.1912372)
Supplement: Supplementary file 1 [file Supplementary_file_1.DOCX]

SUPPLEMENTARY FIGURE S1

Exploratory within-discovery-dataset ROC curves for *Sqstm1*, *Ifngr1*, and *Glul*. The same 14 samples were used for candidate prioritization and ROC assessment; no independent validation cohort was available. The AUC values are descriptive and should not be interpreted as validated diagnostic performance.
