# Supplementary material for: Reduced hepatic GLUL expression accompanies altered RIP3/MLKL-pathway protein abundance in early cholestatic liver injury following bile duct ligation
Source: Front Nutr. 2026 Oct 2;13:1912372. doi: 10.3389/fnut.2026.1912372 (PMC13672626; doi:10.3389/fnut.2026.1912372)
Supplement: Supplementary file 2 [file Table_1.docx]

Supplementary Material

# 1 Supplementary Table

**Table S1**. Primer pairs for RT-qPCR Analysis of Gene Expression

| Name | Forward (5'-3') | Reverse (5'-3') |
| --- | --- | --- |
| SQSTM1 | GGACCCATCTACAGAGGCTG | ACTGGATCGTGTCCAGAGCA |
| IFNGR1 | ACAGCTCTCCGTCCTCGTAT | CACTCCGGTTATGCTCCACA |
| GLUL | AGGCACCAGTACCACATTCG | GGCCGACGGTCTTCAAAGTA |
| MLKL | ATGCCAGCGTCTAGGAAACC | TGTCATCGGGCAGGTTCTTC |
| RIP1 | AACCGCGCTGAGTACAATGA | ATGCCCAGTAGCTTCACCAC |
| RIP3 | ATGTCCTGAGAGGCAAGCAC | TGTCATTGGATTCGGTGGGG |
| GAPDH | GGTTGTCTCCTGCGACTTCA | TGGTCCAGGGTTTCTTACTCC |
